# G3BP1 reduction during cellular senescence promotes SVIL expression and selected senescence-associated phenotypes

**DOI:** 10.3389/fcell.2026.1963301

**Published:** 2026-09-30

**Authors:** Hyeong Bin Jeon, Dahyeon Koh, Dohyang Kim, Chaehwan Oh, Yebin Lee, Minjung Lim, Sanghoon Lee, Ji Yun Han, Jae Hong Yoo, Kyung-Seok Han, Myriam Gorospe, Jaewoo Hong, Ji Heon Noh, Kyoung Mi Kim

**Affiliations:** 1 Department of Biological Sciences, Chungnam National University, Daejeon, Republic of Korea; 2 Institute for Sciences of the Universe, Chungnam National University, Daejeon, Republic of Korea; 3 Department of Physiology, Daegu Catholic University School of Medicine, Daegu, Republic of Korea; 4 Laboratory of Genetics and Genomics, National Institute on Aging Intramural Research Program, National Institutes of Health, Baltimore, MD, United States; 5 CaniCatiCare, Inc., Daegu, Republic of Korea; 6 Molecular Aging Biology Laboratory (MABL), Department of Biochemistry, College of Natural Science, Chungnam National University, Daejeon, Republic of Korea

**Keywords:** cellular senescence, post-transcriptional regulation, Ras GTPase-activating protein-binding protein 1, senescence-associated secretory phenotype, supervillin

## Abstract

RNA-binding proteins (RBPs) can regulate gene expression through multiple post-transcriptional mechanisms, including the control of mRNA stability. In this study, we identified the Ras GTPase-activating protein-binding protein 1 (G3BP1) as a post-transcriptional regulator of *supervillin* (*SVIL*) expression. G3BP1 protein levels were consistently reduced across multiple senescence models, whereas *SVIL* mRNA and SVIL protein levels were increased. RIP analysis showed that *SVIL* mRNA was associated with G3BP1-containing ribonucleoprotein complexes, and G3BP1 depletion in proliferating cells prolonged the half-life of *SVIL* mRNA and increased SVIL expression. In particular, SVIL overexpression increased senescence-associated β-galactosidase (SA-β-gal) activity and selectively elevated a subset of senescence-associated secretory phenotype (SASP)-related mRNAs. These findings suggest that reduced G3BP1 expression during senescence contributes to SVIL upregulation through increased *SVIL* mRNA stability, while elevated SVIL may promote selected senescence-associated phenotypes, including increased expression of SASP-related mRNAs.

## Introduction

Cellular senescence is defined as a state of persistent growth arrest induced by stresses such as telomere shortening, DNA damage, oxidative stress, and oncogene activation (Kuilman et al., 2010; Kumari and Jat, 2021; Kumar and Thirumurugan, 2023; Yamauchi and Takahashi, 2025). Senescent cells exhibit distinct features that distinguish them from proliferating cells, including a flattened and enlarged cell morphology, senescence-associated heterochromatin foci, altered gene expression, and increased senescence-associated β-galactosidase activity (Vicencio et al., 2008; Yanagi et al., 2017; Dodig et al., 2019). Additionally, senescent cells exhibit a senescence-associated secretory phenotype (SASP), characterized by increased expression and secretion of pro-inflammatory cytokines, growth and angiogenic factors, and matrix metalloproteases (Banerjee et al., 2021). While the SASP can play beneficial roles, such as promoting tissue repair, persistent SASP from an increased senescent cell burden can have deleterious effects by promoting chronic inflammation, pathological angiogenesis, and other adverse effects on surrounding cells and systemically (Birch and Gil, 2020; Wilkinson and Hardman, 2020; Sun et al., 2022).

Ras GTPase-activating protein-binding protein 1 (G3BP1) is a multifunctional protein that is highly conserved from yeast to humans. G3BP1 functions as an RNA-binding protein (RBP) and has been shown to regulate the stability of target transcripts, including *MYC* and *PMP22* mRNAs (Parker et al., 1996; Gallouzi et al., 1998; Tourriere et al., 2001; Winslow et al., 2013; Laver et al., 2020). Additionally, G3BP1 associates with highly structured 3′untranslated regions (3′UTRs) of target mRNAs and, together with UPF1, promotes their degradation through structure-mediated RNA decay (SRD) (Fischer et al., 2020). G3BP1 has also been shown to bind RNA G-quadruplexes (rG4s) within target mRNAs and modulate their stability (He et al., 2021). Although G3BP1 has been implicated in stress responses and RNA metabolism, its role in post-transcriptional regulation during cellular senescence remains incompletely understood.

Supervillin (SVIL) is a plasma membrane-associated F-actin-binding protein belonging to the villin/gelsolin superfamily (Pestonjamasp et al., 1997). SVIL participates in cell division, focal adhesion, matrix degradation, and podosome formation (Takizawa et al., 2006; Crowley et al., 2009; Fang et al., 2010; Smith et al., 2010; Bhuwania et al., 2012). In the nucleus, SVIL regulates chromatin metabolism through H3K9 demethylation, and contributes to neuronal maturation (Laurent et al., 2015). SVIL has also been implicated in cancer progression by promoting cancer cell proliferation and survival through inhibition of the tumor suppressor p53. SVIL has further been reported to enhance angiogenesis in liver cancer and to promote metastasis and epithelial–mesenchymal transition in hepatocellular carcinoma via the RhoA/ROCK-ERK/p38 pathway (Fang and Luna, 2013; Chen et al., 2018; Zhao et al., 2020). However, the regulation and function of SVIL during cellular senescence remain unclear.

In this study, we found that G3BP1 protein levels decline across multiple senescence models, whereas *SVIL* mRNA and protein levels increase in senescent cells. We further identified *SVIL* mRNA as a transcript associated with G3BP1-containing ribonucleoprotein (RNP) complexes and showed that G3BP1 negatively regulates *SVIL* mRNA stability in proliferating cells. Functionally, G3BP1 depletion and SVIL overexpression increased SA-β-gal activity and the expression of selected SASP-related mRNAs. These findings suggest that reduced G3BP1 levels during senescence contribute to SVIL upregulation through increased mRNA stability, while elevated SVIL may contribute to selected senescence-associated phenotypes.

## Results

### G3BP1 protein levels decline across multiple senescence models

Proteomic analysis of replicative senescence in WI-38 fibroblasts revealed that G3BP1 protein levels were significantly reduced at late population doubling levels (PDLs) (Chan et al., 2022). G3BP2 also decreased with cellular senescence, but the change was modest compared with G3BP1 (Supplementary Figure S1). To investigate these results in our experimental system, we induced replicative senescence by continuously passaging WI-38 cells. Senescent cells displayed a flattened and enlarged morphology and exhibited a marked increase in senescence-associated β-galactosidase (SA-β-gal) activity (Figure 1a). Consistent with the proteomic analysis, G3BP1 protein levels were significantly reduced in senescent cells compared to proliferating cells (Figure 1b). RT-qPCR analysis revealed that *G3BP1* mRNA levels were slightly but significantly decreased in senescent cells (Figure 1c).

**FIGURE 1:**
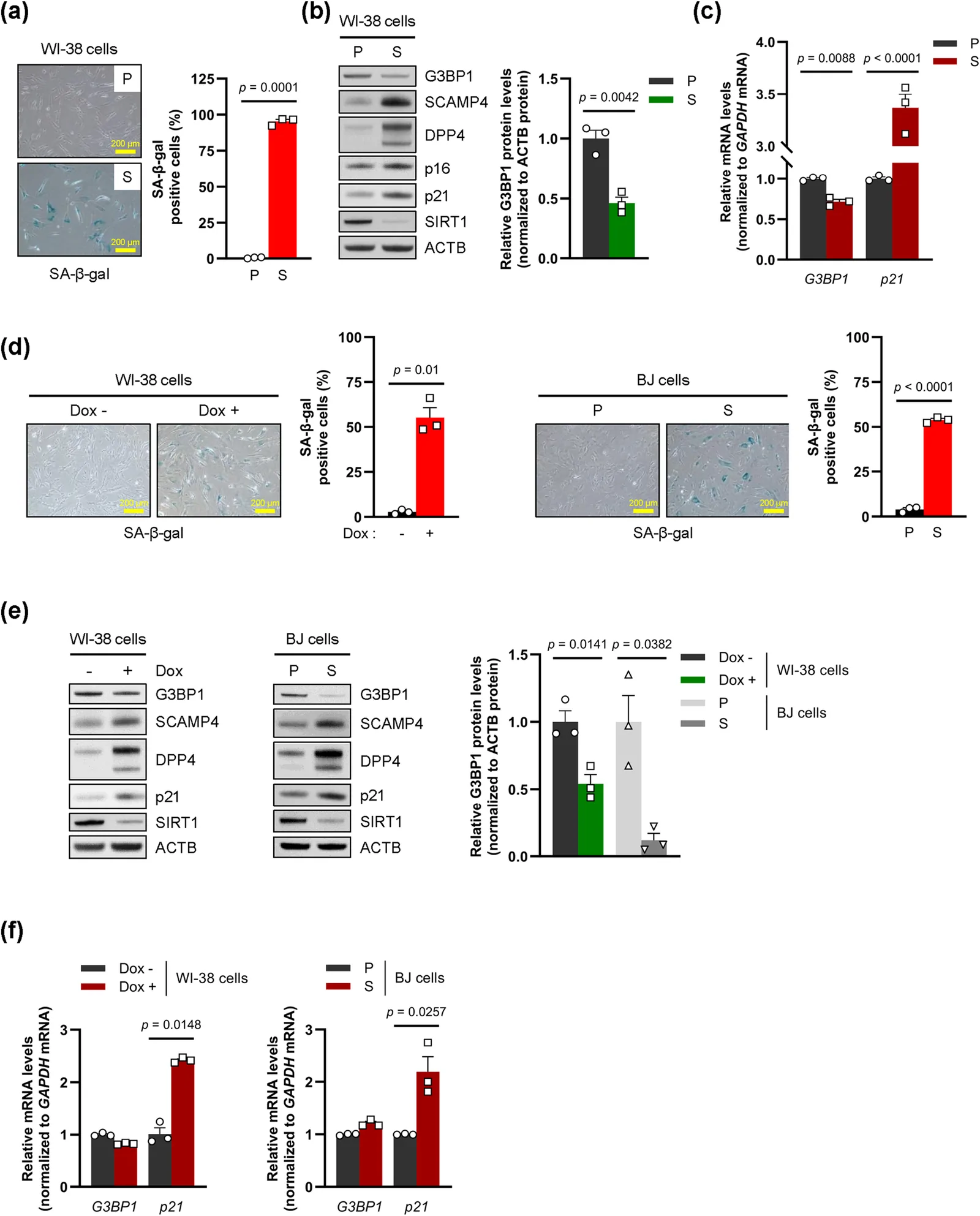
G3BP1 protein levels are reduced in senescent cells. **(a)** Senescence-associated β-galactosidase (SA-β-gal) staining in proliferating (P) and senescent (S) WI-38 cells. SA-β-gal-positive cells were quantified as a percentage of total cells in three randomly selected micrograph fields. **(b)** Western blot analysis of G3BP1 protein and the senescence markers SCAMP4, DPP4, p16, p21 and SIRT1. ACTB was used as a loading control, and protein levels were quantified using ImageJ. **(c)** RT-qPCR analysis of the levels of *G3BP1* mRNA and senescence marker *p21* mRNA, normalized to *GAPDH* mRNA levels. **(d)** SA-β-gal staining in doxorubicin (Dox)-induced senescent WI-38 cells (*left*), and proliferating (P) and replicative senescent (S) BJ cells (*right*). SA-β-gal-positive cells were quantified as a percentage of total cells in three randomly selected micrograph fields. **(e)** Western blot analysis of G3BP1, SCAMP4, DPP4, p21 and SIRT1 in Dox-induced senescent WI-38 cells and in replicative senescent BJ cells. ACTB was used as a loading control, and protein levels were quantified using ImageJ. **(f)** RT-qPCR analysis of the levels of *G3BP1* and *p21* mRNAs, normalized to *GAPDH* mRNA levels. Graphs in **(a–f)** represent the mean ± standard error of the mean (SEM) from three independent experiments. Statistical significance was determined using two-tailed unpaired Welch’s *t*-tests. Exact *p*-values are provided in the figures, with values below 0.0001 indicated as *p* < 0.0001. RT-qPCR, reverse transcription quantitative polymerase chain reaction; P, proliferating; S, senescent; SA-β-gal,senescence-associated β-galactosidase; Dox, doxorubicin; G3BP1, Ras GTPase-activating protein-binding protein 1; SCAMP4, secretory carrier membrane protein 4; DPP4, dipeptidyl peptidase 4; SIRT1, sirtuin 1; ACTB, beta-actin; GAPDH, glyceraldehyde 3-phosphate dehydrogenase.

To study if G3BP1 was reduced in other senescence models, we established stress-induced premature senescence (SIPS) in WI-38 fibroblasts and replicative senescence in BJ fibroblasts. Proliferating WI-38 fibroblasts were treated with doxorubicin (Dox) and cultured for 10 days to induce SIPS, whereas BJ fibroblasts were continuously passaged until replicative exhaustion. Both models exhibited increased SA-β-gal activity (Figure 1d), and Western blot analysis showed reduced G3BP1 protein levels (Figure 1e). However, *G3BP1* mRNA levels did not change significantly (Figure 1f). Thus, *G3BP1* mRNA levels differed among senescence models, whereas G3BP1 protein levels were reduced in all three models examined.

### *SVIL* mRNA is a candidate G3BP1-associated transcript that increases with senescence

Given that G3BP1 is an RBP, we next sought to identify potential target mRNAs using a published G3BP1-associated transcript list derived from enhanced CLIP-seq (eCLIP-seq) analysis (Fischer et al., 2020; He et al., 2021). Because G3BP1 has been implicated in mRNA degradation, we next focused on G3BP1-associated transcripts that were increased in senescent cells (Winslow et al., 2013; Casella et al., 2019; Fischer et al., 2020). Integration of these datasets identified four candidate transcripts: *SVIL*, *AKR1B1*, *CLSTN1*, and *CD59* (Figure 2a). RT-qPCR analysis confirmed that all four candidate mRNAs were significantly increased in senescent WI-38 cells (Figure 2b). We next examined published proteomic datasets to prioritize a candidate for further investigation (Kim et al., 2017; Munk et al., 2021). Among the candidates, SVIL was of particular interest because our previously published cell-surface proteomic analysis showed increased SVIL abundance in senescent cells (Kim et al., 2017). We therefore selected *SVIL* for further investigation of its regulation by G3BP1 and its potential role in senescence-associated phenotypes (Figure 2a).

**FIGURE 2:**
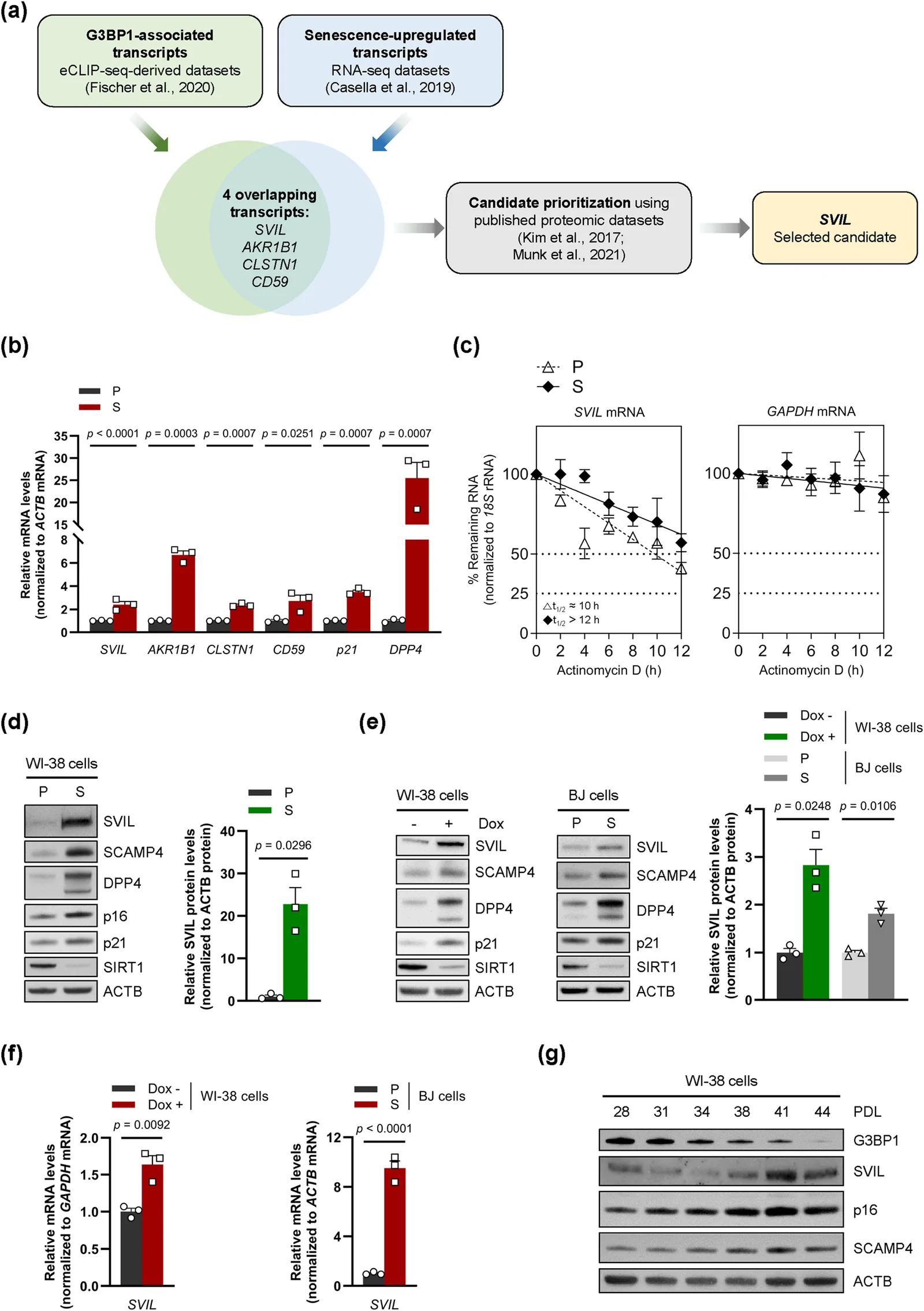
*SVIL* mRNA and protein levels increase in senescent cells. **(a)** Schematic workflow for the identification of G3BP1-associated transcripts upregulated in replicative senescent WI-38 cells and the subsequent prioritization of *SVIL* mRNA. G3BP1-associated transcripts derived from eCLIP-seq datasets were compared with transcripts upregulated in replicative senescent WI-38 cells relative to proliferating cells (log_2_ fold change ≥1 and *p*-value ≤0.01). Four overlapping transcripts were identified: *SVIL*, *AKR1B1*, *CLSTN1*, and *CD59* mRNAs. *SVIL* mRNA was selected for further study based on candidate prioritization using published proteomic datasets. **(b)** RT-qPCR analysis of the levels of *SVIL, AKR1B1, CLSTN1, CD59, p21*, and *DPP4* mRNAs normalized to the levels of *ACTB* mRNA. **(c)** RT-qPCR analysis of the half-lives of *SVIL* and *GAPDH* mRNAs following actinomycin D treatment in proliferating (P) and senescent (S) WI-38 cells, normalized to *18S* rRNA levels. The stable *GAPDH* mRNA served as a negative control. **(d)** Western blot analysis of SVIL, SCAMP4, DPP4, p16, p21 and SIRT1. ACTB was used as a loading control, and protein levels were quantified using ImageJ. **(e)** Western blot analysis of SVIL, SCAMP4, DPP4, p21 and SIRT1. ACTB was used as a loading control, and protein levels were quantified using ImageJ. **(f)** RT-qPCR analysis of *SVIL* mRNA, normalized to *GAPDH* or *ACTB* mRNAs. **(g)** Western blot analysis of G3BP1 and SVIL proteins in WI-38 cells at increasing population doubling levels (PDLs). Senescence markers p16 and SCAMP4 proteins were also analyzed and ACTB was included as a loading control. Graphs in **(b–f)** represent the mean ± standard error of the mean (SEM) from three independent experiments. Statistical significance was determined using two-tailed unpaired Welch’s *t*-tests for **(d–f)** and multiple two-tailed unpaired Welch’s *t*-tests with Holm–Šídák correction for **(b)**. Exact *p*-values are provided in the figures, with values below 0.0001 indicated as *p* < 0.0001. RT-qPCR, reverse transcription quantitative polymerase chain reaction; P, proliferating; S, senescent; Dox, doxorubicin; G3BP1, Ras GTPase-activating protein-binding protein 1; SVIL, supervillin; AKR1B1, aldo-keto reductase family 1 member B1; CLSTN1, calsyntenin 1; CD59, CD59 molecule (CD59 blood group); SCAMP4, secretory carrier membrane protein 4; DPP4, dipeptidyl peptidase 4; SIRT1, sirtuin 1; ACTB, beta-actin; GAPDH, glyceraldehyde 3-phosphate dehydrogenase.

Given that G3BP1 has been reported to promote the degradation of associated mRNAs (Tourriere et al., 2001; Winslow et al., 2013; Fischer et al., 2020; He et al., 2021), we examined whether the higher *SVIL* mRNA levels in senescent cells were associated with increased mRNA stability. We measured *SVIL* mRNA half-life after blocking *de novo* transcription with actinomycin D. The half-life of *SVIL* mRNA was approximately 10 h in proliferating cells but more than 12 h in senescent cells (Figure 2c). This increase in *SVIL* mRNA abundance and stability was accompanied by a rise in SVIL protein levels in senescent WI-38 cells, as determined by Western blot analysis (Figure 2d). Furthermore, SVIL protein and *SVIL* mRNA levels were also increased in Dox-induced senescent WI-38 and replicative senescent BJ cells (Figures 2e,f). To determine whether G3BP1 and SVIL protein levels exhibit opposing patterns during replicative senescence, we analyzed their levels in WI-38 cells at increasing PDLs. G3BP1 protein levels progressively decreased with increasing PDLs, whereas SVIL protein levels markedly increased at late PDLs (Figure 2g). Taken together, these results identify *SVIL* mRNA as a G3BP1-associated candidate transcript and show that *SVIL* mRNA and protein levels are elevated across multiple senescence models.

### *SVIL* mRNA is associated with G3BP1-containing RNP complexes and is negatively regulated by G3BP1

To examine the association between G3BP1 and *SVIL* mRNA, we performed ribonucleoprotein immunoprecipitation (RIP) analysis in proliferating WI-38 cells. *SVIL* mRNA was enriched in the G3BP1 IP compared with the control IgG IP, suggesting that *SVIL* mRNA is associated with G3BP1-containing RNP complexes (Figure 3a). We next used short hairpin RNA (shRNA) to knock down G3BP1 in proliferating WI-38 cells and examined whether G3BP1 depletion affects SVIL expression. G3BP1 depletion increased SA-β-gal activity and resulted in a trend toward increased SVIL and dipeptidyl peptidase 4 (DPP4) protein levels, whereas p53 and p21 protein levels were not markedly altered (Figures 3b,c). RT-qPCR analysis further showed that G3BP1 depletion significantly increased *SVIL* and *p21* mRNA levels (Figure 3d). In addition, G3BP1 depletion significantly increased the levels of the SASP-related interleukin 6 (*IL6)* and C-C motif chemokine ligand 5 (*CCL5*) mRNAs, whereas *IL1A*, *IL1B*, *IL8*, *CCL2*, and *CCL7* mRNAs were not significantly altered (Figure 3e). To determine whether G3BP1 regulates *SVIL* mRNA stability, we next depleted G3BP1 using small interfering RNA (siRNA) and measured *SVIL* mRNA half-life following actinomycin D treatment to block *de novo* transcription. G3BP1 depletion extended the half-life of *SVIL* mRNA from approximately 8 h to more than 12 h, indicating that G3BP1 negatively regulates *SVIL* mRNA stability (Figures 3f,g). These results show that *SVIL* mRNA is associated with G3BP1-containing RNP complexes and that G3BP1 depletion increases *SVIL* mRNA stability and SVIL expression while inducing selected senescence-associated changes.

**FIGURE 3:**
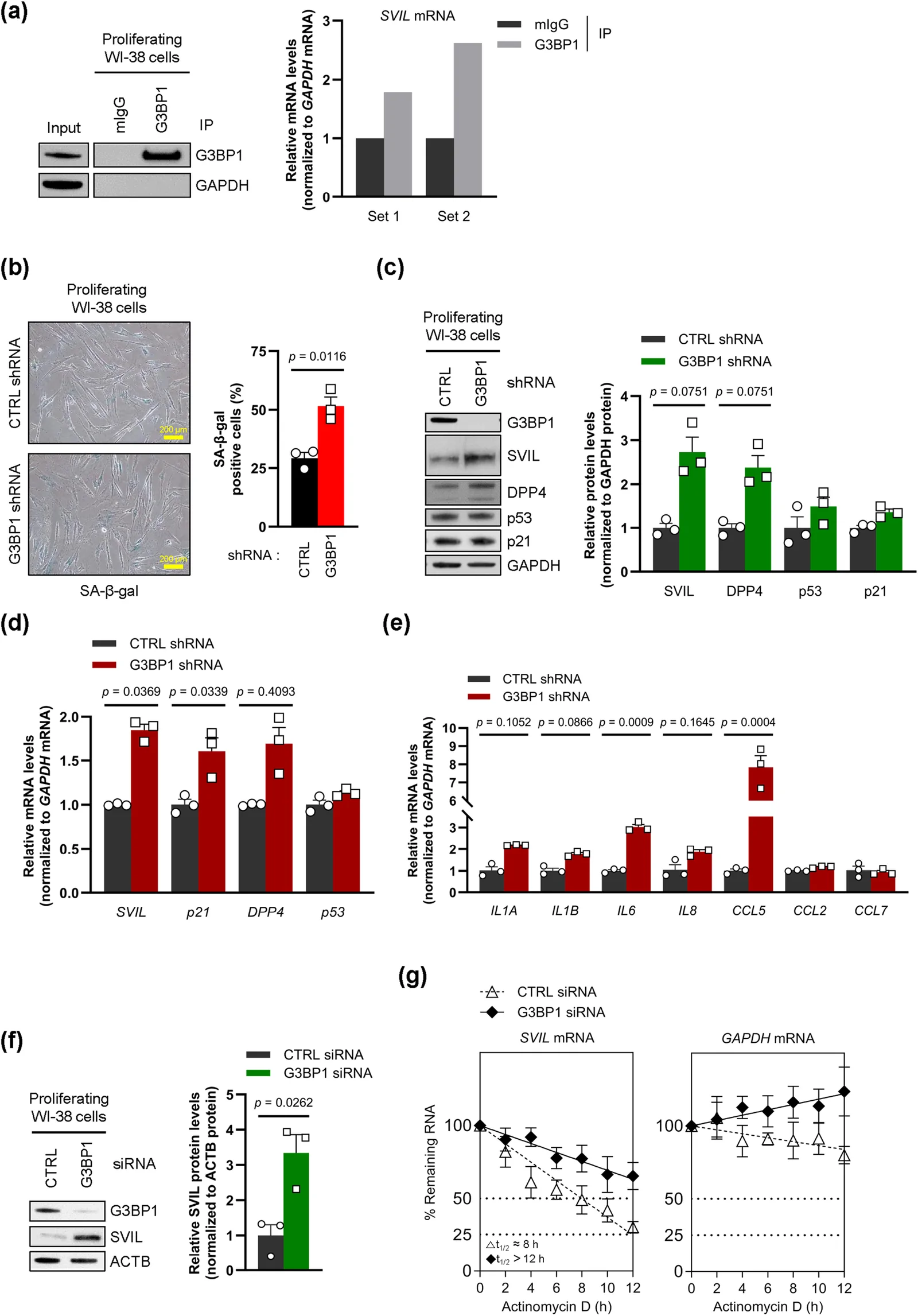
*SVIL* mRNA is associated with G3BP1-containing RNP complexes, and G3BP1 negatively regulates its stability. **(a)** Ribonucleoprotein immunoprecipitation (RIP) analysis of G3BP1 in proliferating WI-38 cells. Immunoprecipitated proteins were assessed by Western blot analysis (*left*), while RIP-enriched *SVIL* mRNA levels were quantified using RT-qPCR analysis and normalized to *GAPDH* mRNA levels. mIgG samples were set to 1 (*right*). The input represents total WI-38 cell lysates prepared in NT2 buffer followed by sonication. Lysates used for IP were probed with anti-G3BP1 and anti-GAPDH antibodies. GAPDH was used as a negative control for IP. **(b–e)** Proliferating WI-38 cells were transduced with CTRL or G3BP1 shRNA-expressing lentiviruses, and all assays were performed 13 days post-transduction. **(b)** SA-β-gal staining. SA-β-gal-positive cells were quantified as a percentage of total cells in three randomly selected micrograph fields. **(c)** Western blot analysis of G3BP1, SVIL, DPP4, p53 and p21. GAPDH served as a loading control. Protein levels were quantified using ImageJ. **(d)** RT-qPCR analysis of the levels of *SVIL, p21, DPP4,* and *p53* mRNAs, normalized to *GAPDH* mRNA levels. **(e)** RT-qPCR analysis of SASP-related mRNAs, normalized to *GAPDH* mRNA. **(f)** Western blot analysis of G3BP1 and SVIL in proliferating WI-38 cells transfected with CTRL siRNA or G3BP1 siRNA. ACTB served as a loading control. Protein levels were quantified using ImageJ. **(g)** RT-qPCR analysis of *SVIL* mRNA and *GAPDH* mRNA half-lives following actinomycin D treatment in proliferating WI-38 cells transfected with CTRL siRNA or G3BP1 siRNA. *GAPDH* mRNA served as a negative control. Graphs in **(b–g)** present the mean ± standard error of the mean (SEM) from three independent experiments. Statistical significance was determined using two-tailed unpaired Welch’s *t*-tests for **(b,f)** and multiple two-tailed unpaired Welch’s *t*-tests with Holm–Šídák correction for **(c–e)**. Exact *p*-values are provided in the figures, with values below 0.0001 indicated as *p* < 0.0001. RT-qPCR, reverse transcription quantitative polymerase chain reaction; SA-β-gal, senescence-associated β-galactosidase; G3BP1, Ras GTPase-activating protein-binding protein 1; SVIL, supervillin; DPP4, dipeptidyl peptidase 4; CTRL, control; shRNA, short hairpin RNA; siRNA, small interfering RNA; IL1A, interleukin 1A; IL1B, interleukin 1B; IL6, interleukin 6; IL8, interleukin 8; CCL2*,* chemokine ligand 2; CCL5, chemokine ligand 5; CCL7, chemokine ligand 7; GAPDH, glyceraldehyde 3-phosphate dehydrogenase ACTB, beta-actin.

### SVIL overexpression increases SA-β-gal activity and selectively elevates SASP-related mRNAs

Since SVIL is elevated in senescent cells, we investigated whether ectopically increasing SVIL levels is sufficient to promote senescence-associated phenotypes in proliferating WI-38 cells. Proliferating WI-38 cells were transduced with lentivirus expressing SVIL or CTRL, and senescence-associated phenotypes were assessed. As shown, SVIL overexpression markedly increased SA-β-gal activity (Figure 4a) and significantly reduced sirtuin 1 (SIRT1) protein levels, whereas DPP4, p16, p21, and secretory carrier membrane protein 4 (SCAMP4) protein levels showed no significant changes (Figure 4b). RT-qPCR analysis further showed that SVIL overexpression selectively increased the SASP-related *IL6*, *IL8*, and *CCL7* *mRNAs*, whereas other SASP-related and senescence marker transcripts were not significantly altered (Figure 4c). These findings suggest that increased SVIL levels contribute to selected senescence-associated phenotypes rather than inducing a broad senescence program.

**FIGURE 4:**
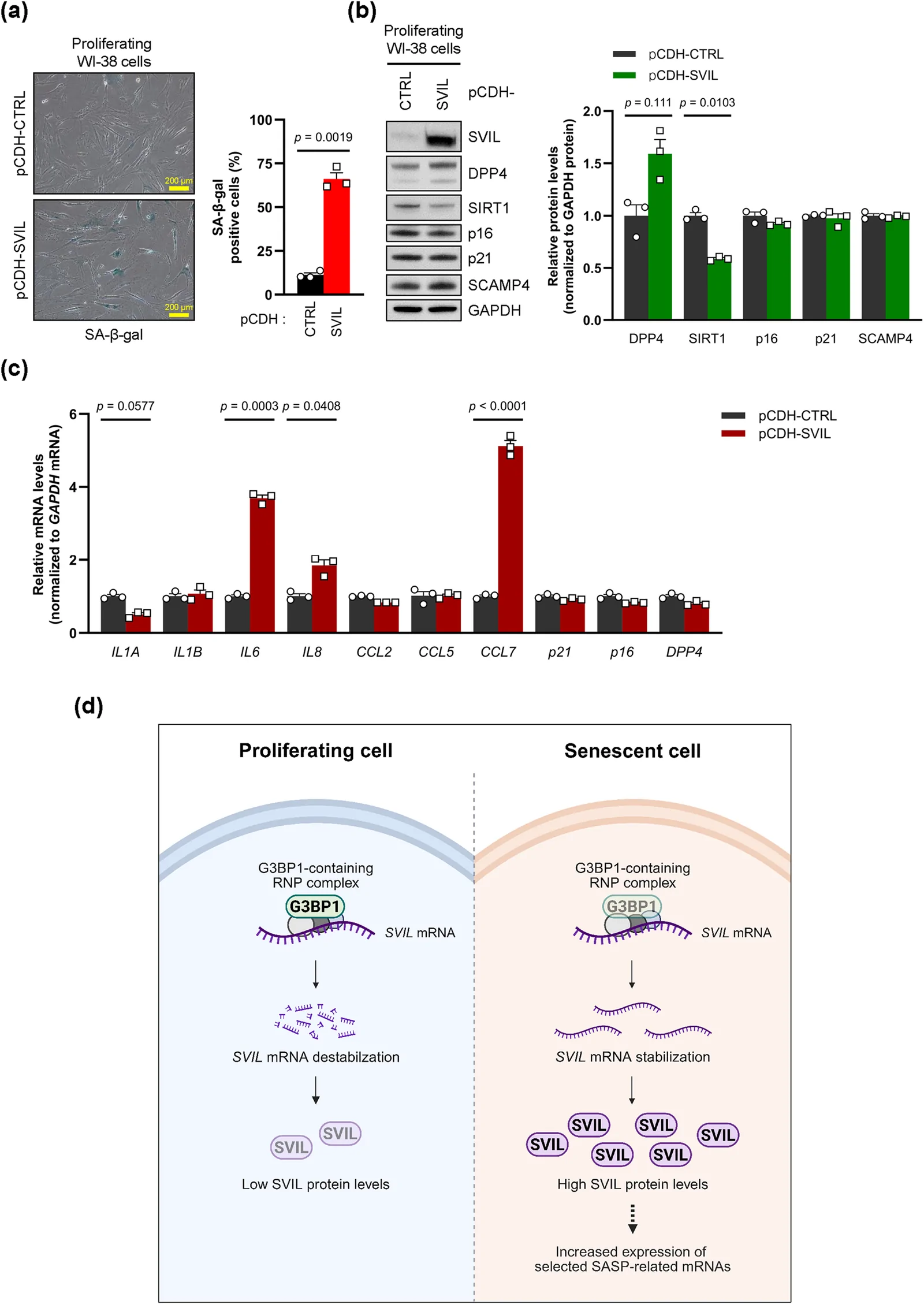
SVIL overexpression increases SA-β-gal activity and selectively elevates SASP-related mRNAs. **(a–c)** Proliferating WI-38 cells were transduced with lentiviruses to express either CTRL or SVIL, and all assays were performed 22 days post-transduction. **(a)** SA-β-gal staining. SA-β-gal-positive cells were quantified as a percentage of total cells in three randomly selected micrograph fields. **(b)** Western blot analysis of SVIL, DPP4, SIRT1, p16, p21, and SCAMP4. GAPDH was used as a loading control, and protein levels were quantified using ImageJ. **(c)** RT-qPCR analysis of SASP-related mRNAs and *p21*, *p16*, and *DPP4* mRNAs, normalized to *GAPDH* mRNA. **(d)** Proposed model**.** In proliferating cells, G3BP1 promotes *SVIL* mRNA destabilization, thereby maintaining low SVIL protein levels. In senescent cells, G3BP1 levels decline, which is associated with increased *SVIL* mRNA stability and elevated SVIL protein. Elevated SVIL protein is accompanied by increased expression of selected SASP-related mRNAs. Graphs in **(a–c)** present the mean ± standard error of the mean (SEM) from three independent experiments. Statistical significance was determined using two-tailed unpaired Welch’s *t*-tests for **(a)** and multiple two-tailed unpaired Welch’s *t*-tests with Holm–Šídák correction for **(b,c)**. Exact *p*-values are provided in the figures, with values below 0.0001 indicated as *p* < 0.0001. RT-qPCR, reverse transcription quantitative polymerase chain reaction; SA-β-gal, senescence-associated β-galactosidase; G3BP1, Ras GTPase-activating protein-binding protein 1; SVIL, supervillin; SCAMP4, secretory carrier membrane protein 4; DPP4, dipeptidyl peptidase 4; SIRT1, sirtuin 1; CTRL, control; IL1A, interleukin 1A; IL1B, interleukin 1B; IL6, interleukin 6; IL8, interleukin 8; CCL2*,* chemokine ligand 2; CCL5, chemokine ligand 5; CCL7, chemokine ligand 7; GAPDH, glyceraldehyde 3-phosphate dehydrogenase; SASP, senescence-associated secretory phenotype RNP, ribonucleoprotein.

## Discussion

RBPs regulate gene expression at multiple post-transcriptional levels during cellular senescence. G3BP1 is a well-characterized RBP involved in post-transcriptional regulation of mRNA stability, but its role in cellular senescence remains less well characterized. In this study, we identified *SVIL* mRNA as a transcript whose expression is negatively regulated by G3BP1 and investigated its potential role in senescence-associated phenotypes. G3BP1 protein levels are reduced across multiple senescence models, including replicative senescent WI-38 fibroblasts, Dox-induced senescent WI-38 fibroblasts, and replicative senescent BJ fibroblasts (Figure 1). In contrast, *SVIL* mRNA and protein levels increased in senescent cells, and *SVIL* mRNA showed greater stability in senescent than in proliferating WI-38 cells (Figure 2). We further found that *SVIL* mRNA is associated with G3BP1-containing RNP complexes and that G3BP1 depletion prolonged *SVIL* mRNA half-life and increased SVIL expression in proliferating cells (Figure 3). G3BP1 depletion also induced selected senescence-associated changes. These findings support a model in which reduced G3BP1 expression during senescence contributes to increased SVIL expression through stabilization of *SVIL* mRNA. SVIL overexpression also increased SA-β-gal activity and selectively enhanced the expression of a subset of SASP-related mRNAs but did not broadly alter other senescence-associated markers (Figure 4).

Although SVIL overexpression reduced SIRT1 protein levels, DPP4 and the canonical senescence markers p16 and p21 showed no significant changes. In addition, only three SASP-related mRNAs, *IL6*, *IL8*, and *CCL7 mRNAs*, were significantly increased by SVIL overexpression. G3BP1 depletion significantly increased the SASP-related *IL6* and *CCL5* mRNAs. Thus, the SASP-related mRNAs affected by G3BP1 depletion only partially overlapped with those increased by SVIL overexpression. These findings suggest that SVIL may contribute to selected senescence-associated phenotypes rather than inducing a complete senescence program. However, the broader senescence-associated changes following G3BP1 depletion cannot be fully attributed to increased SVIL expression. Beyond its effects on SASP-associated gene expression, increased SVIL may also be relevant to other features of senescent cells. Given its known functions as an actin- and myosin II-binding scaffold, SVIL could potentially contribute to the cytoskeletal and morphological changes associated with cellular senescence. Further studies are needed to determine whether increased SVIL directly affects cytoskeletal organization during senescence.

In conclusion, we identified *SVIL* mRNA as a transcript associated with G3BP1-containing RNP complexes and showed that G3BP1 negatively regulates *SVIL* mRNA stability. Our findings suggest that reduced G3BP1 expression during senescence contributes to SVIL upregulation through increased mRNA stability and that elevated SVIL expression selectively influences SASP-associated gene expression. These results identify G3BP1-dependent regulation of *SVIL* mRNA stability as a post-transcriptional mechanism associated with cellular senescence.

## Methods

### Cell culture, senescence induction, and small interfering RNA transfection

Human lung WI-38 fibroblasts and human skin BJ fibroblasts were obtained from Coriell Cell Repositories (Camden, NJ, USA) and the American Type Culture Collection (ATCC, Manassas, VA, USA), respectively. Cells were cultured in Dulbecco’s Modified Eagle’s Medium (DMEM, WELGENE, Gyeongsan, South Korea) supplemented with 10% fetal bovine serum (Gibco, Waltham, MA, USA), 1% antibiotic-antimycotic (Gibco), and 1% non-essential amino acids (Gibco). WI-38 cells were used at PDLs 23–27 for proliferating cells and PDLs 43–52 for senescent cells. BJ cells were used at PDLs 13–15 for proliferating cells and PDLs 33–36 for senescent cells. To elicit stress-induced premature senescence (SIPS), proliferating WI-38 cells were treated with doxorubicin (Dox; 250 nM; Sigma-Aldrich, St. Louis, MO, USA) for 24 h and then cultured for an additional 10 days before harvest for protein and RNA analyses. Senescence-associated β-galactosidase (SA-β-gal) staining was performed in WI-38 and BJ cells using the Senescence-β-Galactosidase Staining Kit (#9860, Cell Signaling Technology, Danvers, MA, USA), following the manufacturer’s instructions. For RNA silencing experiments, proliferating WI-38 cells were transfected with 50 nM control (CTRL) or G3BP1 small interfering RNA (siRNA) (GenePharma, Shanghai, China) using Lipofectamine 2000 (Invitrogen, Waltham, MA, USA) following the manufacturer’s instructions. Cells were analyzed 3 days after transfection. The siRNA sequences used were as follows: CTRL siRNA (5′-UUC​UCC​GAA​CGU​GUC​ACG​U-3′), or G3BP1 siRNA (5′-UCA​ACA​UGG​CGA​AUC​UUG​G-3′).

### Plasmid construction, lentivirus generation, and cellular transduction

To construct the pSicoR-mCh-G3BP1 shRNA plasmid, and shRNA targeting *G3BP1* mRNA was designed based on a previously reported sequence (Zhang et al., 2021) and synthesized (Bioneer, Daejeon, South Korea). The G3BP1 shRNA sequence was 5′-GGA​TTG​GAT​TCA​AAT​GGA​A-3′. The G3BP1 shRNA oligonucleotides were annealed and cloned into the XhoI and HpaI sites of the pSicoR-mCherry plasmid. The sequence of the constructed plasmid was verified by Sanger sequencing (Macrogen, Seoul, South Korea).

To construct the pCDH-SVIL plasmid, the human coding sequence of *SVIL* mRNA was subcloned from EGFP-supervillin (Addgene plasmid #13040; Watertown, MA, USA) into the BamHI and SalI sites of the pCDH-CMV-MCS-EF1-Puro lentiviral vector (System Biosciences, Palo Alto, CA, USA). The insert was amplified using the following primers: 5′-AAT​TTA​AAT​CGG​ATC​CAT​GAA​AAG​AAA​AGA​AAG​AAT​TGC​CA-3′ (forward) and 5′-GAG​GTT​GAT​TGT​CGA​CCT​AGA​ACA​GGC​CTT​TTG​CTT​TC-3′ (reverse). Gel-purified PCR products (Cosmo Genetech, Seoul, South Korea) were ligated into pre-digested pCDH-CMV-MCS-EF1-Puro. The sequence of the constructed plasmid was verified by Sanger sequencing (Cosmo Genetech or Bioneer).

Lentiviruses expressing SVIL, G3BP1 shRNA, or the corresponding controls were generated by co-transfecting HEK293T cells (ATCC) in 60-mm culture dishes with the appropriate transfer plasmid, packaging plasmid, and envelope plasmid. HEK293T cells were cultured in DMEM (WELGENE) supplemented with 10% fetal bovine serum (Gibco), 1% antibiotic-antimycotic (Gibco). Twenty-four hours after transfection, culture supernatants were collected and centrifuged to remove cell debris. To generate G3BP1-depleted WI-38 cells, proliferating WI-38 cells were transduced with G3BP1 shRNA-expressing or control shRNA lentiviruses with 8 μg/mL of polybrene (Santa Cruz Biotechnology, Dallas, TX, USA). The medium was replaced with fresh medium 24 h after transduction. Cells were harvested 13 days post-transduction for subsequent analyses. To generate SVIL*-*overexpressing WI-38 cells, proliferating WI-38 cells were transduced with SVIL-expressing or control lentiviruses with 8 μg/mL of polybrene (Santa Cruz Biotechnology). The medium was replaced with fresh medium 24 h after transduction. Cells were harvested 22 days post-transduction for subsequent analyses.

### RNA extraction and RT-qPCR analysis

Total RNA was extracted using TRI Reagent™ (Invitrogen) and treated with DNase I (Thermo Fisher Scientific, Waltham, MA, USA). cDNA was synthesized using dNTPs, random hexamers, and RevertAid reverse transcriptase (Thermo Fisher Scientific). qPCR was performed using KAPA SYBR® FAST qPCR Master Mix (Roche, Basel, Switzerland) and a QuantStudio 1 Real-Time PCR System (Thermo Fisher Scientific). Specific primers with sequences in the sense and antisense orientation to amplify the corresponding mRNAs are listed: 5′-TAA​TCG​CCT​TCG​GGG​ACC​TG-3′ and 5′-AAG​CCC​CCT​TCC​CAC​TCC​AA-3′ for *G3BP1*, 5′-CAC​GGT​TGC​TCC​AAT​GTA​TG-3′ and 5′-CTC​TAC​CAA​AGG​CTG​CCA​AG-3′ for *SVIL*, 5′-GAT​CGC​AGC​CAA​GCA​CAA​TA-3′ and 5′-CTT​GTG​GGA​GGT​ACA​GCT​CA-3′ for *AKR1B1*, 5′-TGA​TGA​GGA​ATG​GCA​CCA​CT-3′ and 5′-CCT​GAA​AAC​TCT​TGC​CAG​CA-3′ for *CLSTN1*, 5′-TGG​CTC​CCA​CAG​TTA​GAA​GG-3′ and 5′-AGA​CTC​CAG​GTT​CTT​CAG​CC-3′ for *CD59*, 5′-AGT​GCT​GCT​GAA​GGA​GAT​GCC​TGA-3′ and 5′-CCC​CTG​CCA​AGC​ACA​CCC​AGT​A-3′ for *IL1A*, 5′-TGC​ACG​CTC​CGG​GAC​TCA​CA-3′ and 5′-CAT​GGA​GAA​CAC​CAC​TTG​TTG​CTC​C-3′ for *IL1B*, 5′-CCA​GGA​GCC​CAG​CTA​TGA​AC-3′ and 5′-CCC​AGG​GAG​AAG​GCA​ACT​G-3′ for *IL6*, 5′-GAG​TGG​ACC​ACA​CTG​CGC​CA-3′ and 5′-TCC​ACA​ACC​CTC​TGC​ACC​CAG​T-3′ for *IL8*, 5′-CCT​TCT​GTG​CCT​GCT​GCT​CAT​AG-3′ and 5′-TCT​TCG​GAG​TTT​GGG​TTT​GCT​TGT-3′ for *CCL2*, 5′-CCA​TAT​TCC​TCG​GAC​ACC​AC-3′ and 5′-GGG​TGA​CAA​AGA​CGA​CTG​CT-3′ for *CCL5*, 5′-CTC​TGC​AGC​ACT​TCT​GTG​TC-3′ and 5′-GGA​CAG​TGG​CTA​CTG​GTG​GT-3′ for *CCL7*, 5′-CGG​TCG​GAG​GCC​GAT​CCA​G-3′ and 5′-GCG​CCG​TGG​AGC​AGC​AGC​AGC​T-3′ for *p16,* 5′-CTG​CCC​AAG​CTC​TAC​CTT-3′ and 5′-AAG​GCA​GAA​GAT​GTA​GAG​C-3′ for *p21*, 5′-CCC​CAG​CCA​AAG​AAG​AAA​C-3′ and 5′-AAC​ATC​TCG​AAG​CGC​TCA​C-3′ for *p53*, 5′-CAA​ATT​GAA​GCA​GCC​AGA​CA-3′ and 5′-CAC​ACT​TGA​ACA​CGC​CAC​TT-3′ for *DPP4*, 5′-TGC​ACC​ACC​AAC​TGC​TTA​GC-3′ and 5′-GGC​ATG​GAC​TGT​GGT​CAT​GAG-3′ for *GAPDH*, 5′-CGA​ACG​TCT​GCC​CTA​TCA​ACT​T-3′ and 5′-ACC​CGT​GGT​CAC​CAT​GGT​A-3′ for *18S* rRNA, and 5′-CAT​GTA​CGT​TGC​TAT​CCA​GGC-3′ and 5′-CTC​CTT​AAT​GTC​ACG​CAC​GAT-3′ for *ACTB*.

The stability of mRNA was assessed by treating cells with 2 μg/mL actinomycin D (Sigma-Aldrich), followed by measurement of mRNA decay over time to determine mRNA half-lives.

### Protein extraction, Western blotting, and ribonucleoprotein immunoprecipitation (RIP) analyses

Cells were lysed using M-PER^TM^ Mammalian Protein Extraction Reagent (Thermo Fisher Scientific) supplemented with Halt™ Protease and Phosphatase Inhibitor Cocktail (Thermo Fisher Scientific). Protein lysates were electrophoresed on a sodium dodecyl sulfate (SDS)-polyacrylamide gel (NuPAGE™ 4%–12% Bis-Tris Protein Gels, Invitrogen) and transferred to nitrocellulose membranes (iBlot®2 NC regular stack, Invitrogen) for detection using the following primary antibodies: DPP4 (67,138) from Cell Signaling Technology; GAPDH (sc-32233), G3BP1 (sc-365338), p16 (sc-56330), p53 (sc-126), SVIL (sc-53556), ACTB (sc-47778) from Santa Cruz Biotechnology; SCAMP4 (ab10647) and SIRT1 (ab32447) from Abcam (Cambridge, UK); p21 (05-345) from Millipore (Burlington, MA, USA); p16 (551154) from BD Biosciences (Franklin Lakes, NJ, USA). After incubation with horseradish peroxidase-conjugated secondary antibodies (Millipore), signals were detected with KwikQuant Ultra Digital-ECL Solution (Kindle Biosciences, Greenwich, CT, USA) and imaged with KwikQuant Imager (Kindle Biosciences). Protein levels were quantified using ImageJ software.

For ribonucleoprotein immunoprecipitation (RIP) analysis, proliferating WI-38 cells were lysed using NT2 buffer (50 mM Tris-HCl [pH 7.5], 150 mM NaCl, 1 mM MgCl_2_, 0.05% NP40, 0.1 mM DTT) supplemented with Ribolock RNase inhibitor (Thermo Fisher Scientific) and Halt™ Protease and Phosphatase Inhibitor Cocktail (Thermo Fisher Scientific). Following sonication (70% amplitude, 1 s on/1 s off, 60 cycles, Fisher Scientific, Waltham, MA, USA) and centrifugation at 9,500 rpm for 10 min at 4 °C, the supernatant was used for immunoprecipitation. Primary antibodies against G3BP1 or normal mouse IgG were prebound to rProtein A/G agarose 4B (T&I, Chuncheon, South Korea). The supernatant was then incubated with the antibody-prebound beads with rotation for 2 h at 4 °C. The immunoprecipitated proteins were then washed with NT2 buffer and resuspended in NuPAGE™ LDS Sample Buffer (Thermo Fisher Scientific). Prior to RNA extraction, proteins were removed using proteinase K (20 mg/mL; Enzynomics, Daejeon, South Korea) and 0.05% SDS and then the RNA was extracted using acidic phenol/chloroform treatment. The RNA was then reverse-transcribed and analyzed by qPCR.

### Analysis of publicly available RNA-seq data

Publicly available RNA-seq data from proliferating and replicative senescent WI-38 cells were obtained from GEO (accession number GSE130727; https://doi.org/10.1093/nar/gkz555). This dataset contains two independent biological replicates per condition. We applied filtering criteria of log_2_ fold change ≥ 1 and *p*-value ≤ 0.01 to identify candidate genes for downstream analyses. G3BP1-associated transcripts were obtained from the transcript list provided in the supplementary information of Fischer et al. (2020), which was derived from eCLIP-seq analysis of ENCODE G3BP1 eCLIP-seq data (accession number ENCSR721HPX). The overlap between senescence-upregulated transcripts and G3BP1-associated transcripts was used to identify candidate genes for downstream analyses.

### Public proteomic data analysis

Previously published mass spectrometry-based proteomic data were used to assess the expression levels of G3BP1 and G3BP2 in senescent cells. Specifically, protein expression values for G3BP1 and G3BP2 were extracted from a published proteomic dataset of WI-38 human lung fibroblasts collected at increasing PDLs (n = 3) (Chan et al., 2022). Log_2_ fold changes and adjusted *p*-values were summarized in Supplementary Figure S1.

## Statistical analysis

Most experiments were conducted with three biologically independent replicates. Statistical comparisons between two independent groups were performed using two-tailed unpaired Welch’s t-tests. For experiments involving multiple comparisons, the Holm–Šídák method was used to correct for multiple testing. Results are expressed as the mean ± SEM. Exact *p*-values are provided in the figures, with values below 0.0001 indicated as *p* < 0.0001.

## Data Availability

The raw data supporting the conclusions of this article will be made available by the authors, without undue reservation.
