# Supplementary material for: G3BP1 reduction during cellular senescence promotes SVIL expression and selected senescence-associated phenotypes
Source: Front Cell Dev Biol. 2026 Sep 30;14:1963301. doi: 10.3389/fcell.2026.1963301 (PMC13661574; doi:10.3389/fcell.2026.1963301)
Supplement: Supplementary file 1 [file Table1.docx]

**SUPPLEMENTAL FIGURE LEGENDS**

**Fig. S1. G3BP1 and G3BP2 protein changes derived from published supplementary proteomic data.** G3BP1 and G3BP2 protein expression values were derived from the supplementary information of a previously published mass spectrometry-based study of WI-38 human lung fibroblasts at increasing population doubling levels (PDLs) (Chan et al., 2022). The table summarizes log_2_ fold changes from each PDL (PDL25, 29, 33, 37, 46, and 50) relative to PDL20.

**REFERENCES**

Chan, M., Yuan, H., Soifer, I., Maile, T.M., Wang, R.Y., Ireland, A., et al. (2022). Novel insights from a multiomics dissection of the Hayflick limit. *Elife* 11. doi: 10.7554/eLife.70283.
